# Conceptualisation of a measurement framework for Needs-based Quality of Life among patients with multimorbidity

**DOI:** 10.1186/s41687-022-00489-0

**Published:** 2022-07-27

**Authors:** Kristine Bissenbakker, Anne Møller, John Brandt Brodersen, Alexandra Brandt Ryborg Jønsson

**Affiliations:** 1 Research Unit for General Practice in Copenhagen, Department of Public Health, University of Copenhagen, Copenhagen, Denmark; 2 The Section of General Practice, Department of Public Health, University of Copenhagen, Copenhagen, Denmark; 3 The Research Unit for General Practice in Region Zealand, Copenhagen, Denmark; 4 Department of People and Technology, Roskilde University, Roskilde, Denmark

**Keywords:** Quality of life, Multimorbidity, Patient-reported outcome measure, Needs-based approach

## Abstract

**Background:**

Multimorbidity is both an individual and societal problem. For society, patients with multimorbidity increase healthcare costs. For the individual, living with multimorbidity is complex, and there is an inverse relationship between a patient's Quality of Life (QoL) and their number of chronic conditions. Numerous intervention studies target these problems, yet there is no multimorbidity-specific patient-reported outcome measure (PROM) developed specifically for this group with adequate measurement properties to assess QoL. This study explores what overall needs regarding QoL are affected by living with multimorbidity through qualitative interviews. With this, we conceptualise Needs-based QoL specifically for this group, ensuring high content validity (regarding relevance and comprehensiveness) of using the Needs-based approach to measure their QoL. This is essential as this preliminary study leads to the development of the MultiMorbidity Questionnaire (MMQ), a PROM measuring QoL among patients with multimorbidity.

**Methods:**

This study draws upon qualitative interviews with fifteen patients with multimorbidity based on a semi-structured interview guide following the Needs-based approach. This approach allowed the patients to cover needs relevant for their QoL in relation to the complexities of living with multimorbidity. The transcribed interviews were thematically analysed, inspired by Braun and Clarke’s reflexive approach.

**Results:**

Analysis of the interviews resulted in the construction of six intertwined domains relevant to patients with multimorbidity, covering their Needs-based QoL; “Physical ability”, “Self-determination”, “Security”, “Partner and social life”, “Self-image”, and “Personal finances”. “Physical limitations” and “Personal finances” were stated as core needs implicating the other domains.

**Conclusion:**

This study shows six intertwined overall domains relevant for patients with multimorbidity regarding their Needs-based QoL; “Physical ability”, “Self-determination”, “Security”, “Partner and social life”, “Self-image”, and “Personal finances”. These needs are relevant in a Danish context, with a generally high standard of living. Based on this conceptual framework of Need-based QoL for patients with multimorbidity, we will develop items for a new patient-reported outcome measure called the MMQ.

**Supplementary Information:**

The online version contains supplementary material available at 10.1186/s41687-022-00489-0.

## Background

The number of adults living with multimorbidity, two or more chronic diseases, is rising [1]. The prevalence of multimorbidity increases with age and socioeconomic deprivation [2, 3], and demographics demonstrate that the number will increase further in the future. From a societal perspective, multimorbidity increases healthcare costs because of the patients' extended healthcare use [4]. For the individual, multimorbidity is associated with lower self-reported *Health-Related Quality of Life* (HRQoL) [5–9]. Qualitative studies show how living with multimorbidity influences relationships, social- and working life [5, 8, 10] and how patients struggle to obtain a meaningful everyday life [5]. Several intervention studies aim toward solving these societal and individual problems; still, when focusing on aspects of quality of life (QoL), there is a high degree of heterogeneity in both the utilised outcome measures and the measured effects of the studies [8, 11–14].

### Existing measures of quality of life for patients with multimorbidity

The term QoL moved into the medical sphere with the apparent success of technical treatment in the 70 s, which raised the discussion of prolonged lives *"only at considerable sacrifice of quality of life"* [15]. Simple survival data were no longer enough for the rising population living with chronic illnesses [15]. Hence, QoL became a legitimate clinical medical outcome, despite disagreement on defining QoL and the lack of a standardised measurement tool [16]. The term QoL is still poorly defined and takes on different meanings according to the application area [17, 18]. QoL measures in clinical medicine and clinical trials are frequently linked to the term *health* and focus on aspects related to disease or treatment [17, 19]*.* HRQoL measurement refers to the impact of clinical conditions on functional capacity by primarily assessing the capacity to perform usual daily roles and activities. This functionalist approach is useful from a clinical perspective [20, 21] but has limitations when observed from the patients' perspective. Living with multimorbidity is complex for some patients because of﻿ the influencing social, personal and societal factors [5, 22, 23]. If these complexities are not incorporated in HRQoL measures, they assume that deviation from norm values results in reduced HRQoL [24]; for example, by assuming that patients with chronic limitations in functional capacity or unemployed people have decreased QoL [20].

EQ-5D and SF-36 are the most frequently used HRQoL instruments used in intervention studies for patients with multimorbidity [6, 7, 9, 25]. Both measures are developed without direct patient input [26], and both are examples of generic measures that assess HRQoL irrespective of the underlying diseases [27]. Generic measurement is problematic if a subgroup, for example a diagnostic or cultural group, responds differently to a specific item, a problem referred to as differential items functioning (DIF) [28]. The risk of DIF applies to all patients with chronic illnesses; still, our hypothesis is that it is especially high among patients with multimorbidity due to their heterogenicity. The different combinations of diagnoses lead to different impacts on health, symptoms, impairment, severity, and prognoses.

Yet, despite the risk of DIF when using generic measurement among patients with multimorbidity and the lack of a universally accepted and clear operational definition for the conceptualisation of QoL, it is regarded as an essential core outcome in quantitative studies for this group [29]. This points to the pivotal question: how is QoL measured among patients with multimorbidity in quantitative studies?

We systematically reviewed existing patient-reported outcome measures (PROMs) targeted patients with multimorbidity and assessed the identified measures' development, content validity, structural validity, and internal consistency [30]. The assessment was done following the Consensus-based Standards for Selection of health Measurement INstruments (COSMIN), guidelines for assessing the methodological quality of individual PROM's measurement properties [31]. Surprisingly, none of the identified PROMs included in our systematic review focused on QoL, but on difficulties in encounters with the healthcare system, performing healthcare tasks, the burden of treatment and illness perceptions. Furthermore, none of the identified PROMs possessed adequacy in measurement properties [30].

### The Needs-based approach as a conceptual framework

Since 1992, over thirty PROMs based on the Needs-based approach to QoL have been developed for single diseases, for example, depression, osteoarthritis, asthma and Crohn’s disease [32–35]. These PROMs have shown to be more responsive as outcome measures in clinical studies than generic measurements [36]. Hunt and McKenna operationalised the Needs-based approach to QoL with the aim to develop outcome measures built on what is relevant to the patient with a specific disease [35]. The approach is based on the assumption that life gains quality from a person’s ability to achieve their goals and fulfil their needs [35, 37]. This definition of QoL can be dated back to Thomas More at the beginning of the 1900s, who described basic needs as necessary for human life quality [38]. Later Maslow used the needs theory to describe human motivation and a hierarchy of five human needs: Physiological, Safety, Belongingness, Esteem, and Self-actualization [39]. Humans are motivated by the desire to achieve or maintain these needs, and successful fulfilment of the needs is hypothesised to increase QoL [35, 39, 40]. The Needs-based approach to QoL moves beyond the traditional functional perspective of HRQoL measures to a patient perspective, as function is only relevant if it enables particular needs to be fulfilled [41]. Therefore, in the development process of PROMs with the Needs-based approach, much attention is given to the item generation through qualitative interviews. It is a requirement that patients in the target group are allowed to talk beyond symptoms and loss of functionality and are interviewed openly on how their disease and treatment hinder them in fulfilling needs in their daily life [42]. Thus, the Needs-based approach to developing measurement instruments has a greater patient involvement and a holistic approach to the patient compared to the widely used generic instruments [20]. To our knowledge, no PROM measuring QoL using the Needs-based approach as the framework has targetted patients with multimorbidity [30]. We hypothesise that items in a PROM constructed with the Needs-based approach are less prone to possess DIF since the items cover the basic needs of all humans, thereby taking the heterogeneity and the complexity of living with multimorbidity into account.

Therefore, we plan to develop a PROM for measuring Needs-based QoL among patients with multimorbidity explicitly conceptualised for this group: the MultiMorbidity Questionnaire (MMQ).

This study focuses solely on the conceptual framework behind the construct of interest, QoL, as it is essential in developing a PROM [24, 43, 44]. The conceptual framework is the model or theory, in this case, the Needs-based approach used to frame and describe—and thereby define the construct of interest, QoL [45].

Hence, this study explores which overall needs regarding QoL are affected by living with multimorbidity through qualitative interviews. As a result of this, we conceptualise Needs-based QoL specifically for this group which ensures high content validity (regarding relevance and comprehensiveness) of using the Needs-based approach to measure their QoL.

## Methods

### Content validity of the framework

Content validity is regarded as the most crucial measurement property of a PROM by the COSMIN initiative [46, 47]. This refers to whether a clear description is provided, so that it is well-defined what the final PROM intends to measure [46]. Content validity is obtained with the Needs-based approach through qualitative interviews that allow the informants to dictate the interview direction and raise issues they consider necessary for their QoL as the first step to developing a PROM [42].

### Study design and setting

Interviews with 15 informants with multimorbidity living on Zealand, Denmark, were carried out from November 2018 to September 2019. Four of the participating patients were interviewed twice, as preliminary analysis demonstrated new themes relevant to explore, adding up to ﻿19 qualitative interviews in total. To interview the informants in their natural setting, all interviews were conducted in their private homes except for one, who preferred it to take place at his GP's clinic. Table 1 shows the participating informants' characteristics. The cited informants are used as key informants as their particular articulations coin more general perspectives. For illustrative examples from multiple interviewees, see Table 2. The author group consists of three medical doctors (two of them specialised GPs) and one anthropologist.

**Table 1:** Characteristics of informants–characteristics are altered to secure anonymity

Name	Sex	Age	Marital status	Chronic health problems	Education and occupation
Arthur*	M	84	Married	Chronic obstructive pulmonary disease (COPD), sclerosis, heart failure, glaucoma, depression, osteoarthrosis, high blood pressure	Farming: Retired
Bertha*	F	81	Widow	Asthma, diabetes, osteoarthritis	Clerical background: Retired
John*	M	72	Single	Heart failure, diabetes	Academic: Retired
Frida*	F	69	Married	Diabetes, high blood pressure	Social assistant: Retired
Marco*	M	76	Married	Terminal pancreas and bladder cancer with dissemination, COPD, heart failure, high blood pressure	Teaching: Retired
Carl*	M	77	Separated	COPD, heart failure, diabetes, osteoporosis, osteoarthritis	Draughtsman: Retired
Mary	F	59	Separated	Bipolar, diabetes, migraine	Social assistant: Part-time employment
Robert	M	52	Separated	Diabetes, reduced vision, neuropathy in feet, osteoarthritis, chronic pain in hips, liver cirrhosis, depression	Self-employed clerical background: Early retirement
Daniel	M	48	Partner	Diabetes, hypothyroidism, ADHD	Workman: Part-time lightweight duties
Laura	F	39	Single	Schizophrenia, psoriasis, post-traumatic stress disorder (PTSD)	Unskilled: Early retirement
James	M	52	Married	Chronic back pain after a traffic accident, depression, anxiety, PTSD	Workman: Early retirement
Thomas	M	61	Married	Depression, chronic back pain, osteoporosis, COPD, asthma, heart failure, cataracts, bladder control problems	Service-sector: Early retirement
Peter	M	49	Partner	ADHD, depression, anxiety, herniated disc with chronic back pain after job-related accident, psoriasis arthritis	Unskilled labourer: Early retirement
Amanda	F	69	Single	Colitis ulcerous, osteoarthritis, depression, general pain, hidrasadenitis suppurativa, (chronic skin condition with abscesses)	Unskilled: Retired
William	M	60	Separated	Osteoarthritis, heart failure, physical limitations after cerebral thrombus, herniated disc, asthma, COPD, eczema	Unskilled labourer: Early retirement

^*^First round of interviews before specified inclusion criteria. Therefore, the risk factor high blood pressure is included among these informants

**Table 2:** Descriptions of needs affected within each domain

	Overall descriptions of needs	Example of informant quotations
**Physical ability**	Physically limited in overcoming activities such as personal hygiene, domestic duties, physical activities for the pleasure of it. Push themselves physically to keeping active	*“…some days you just lie here and can’t do a bloody thing, and there I just have to say it is damn irritating because you want to do so many things. I just don’t have the strength […]. If I have to get something from the other end of the store, I don’t have enough breath for it” (William, 60)*
**Self-determination**	Limited by planning around diseases, hindered in impulsive activities, feels depended on others, limited to one’s home or hindered in hobby activities. Need help in everyday life	*“I’m limited by illness, pain, and then the system. Because there are things I’m not allowed to do, things I cannot do, things I shouldn’t do, things I have to do and so on. I want to be independent of all that and be able to take care of myself” (Robert, 52)*
**Security**	Worries about the future or of being a burden to relatives because of insecurities about health conditions and/or treatment. Push themselves mentally	*“I have worries regarding my illnesses. And it is not the separate illnesses, but the combination of them. What I’m mostly concerned about is the illnesses that have to function together and the two kinds of medicine” (Mary, 59)*
**Partner and social life**	Illnesses limit social gatherings, feel as a burden to others, needs support from network, limited in getting new acquaintances. No energy to support others mentally	*“I was […]in the airport at night, and then I drove home with the chauffeur again. And that was damn sad watching the others leave and then going home again” (Daniel, 48, explaining how he had to stay at home from the annual trip abroad with his friends because of his poorly controlled diabetes)*
**Self-image**	Embarrassed about limitations because of health conditions, affected self-esteem, feel stereotyped, blame themselves, bad conscience because of lifestyle	*“I have been very hysterical about my home having to be clean. I cannot overcome that anymore. It bothers me […]. I’m not proud having guests over. Then I feel embarrassed. […] It has something to do with ones vanity.” (Laura, 39)*
**Personal finances**	Illnesses limit means, worries about finances or status in the society	*”And not having money, not being able to buy clothes, not being able to travel anywhere, wearing clothes held together by gaffer tape. I think it is a mental burden […]. It’s the insecurity, can I go on living here?” (Mary, 59)*

### Participants

The informants were patients with multimorbidity (hereafter referred to as informants) recruited by general practitioners (GPs). Informants' identities, including their names and occupation, are altered for anonymity. The inclusion criteria were informants over 18 living with more than one chronic illness (could be both somatic or mental). Furthermore, the informants were sampled with variation regarding gender, age, diagnoses, marital status, rural or urban areas and level of education to obtain central themes relevant across the diverse sample [48]. After the first six interviews, it was necessary to specify the inclusion criteria as the first sample included little variation and specificity [49], for example, an informant experiencing no flaws in QoL with her well-regulated type 2 diabetes and high blood pressure [50]. The specified inclusion criteria were a purposive sample choice [48] to ensure information power and to guide sample size according to our specific aim [49] by including information-rich informants experiencing limitations in their QoL. Having the future development of MMQ in mind, it was necessary to encompass patients who experience the complexity of living with multimorbidity; as our hypothesis is, this group would have the most effect from an intervention study. The specifications of the inclusion criteria were based on knowledge of the impact on HRQoL of specific patterns of multimorbidity [51] as well as socioeconomic class and mental disorders association with multimorbidity [2]. This was concordant with the author group’s clinical experience and empirical knowledge of the patient group. Furthermore, we strived to include informants with the common diagnoses: coronary heart disease, diabetes, chronic obstructive pulmonary disease (COPD) and depression [2]. The broad age range (39–84) of sampled informants encompasses knowledge that the number of chronic diseases increases with age [1], but the absolute number of patients with multimorbidity is highest under the age of 65 years [2]. The specifications included, for example, age under 65 years and/or one or more psychiatric diseases and/or a compromised social network. (Specified inclusion criteria see Additional file 1). Furthermore, the specifications led us to define multimorbidity in our study by the criteria suggested by Willadsen [52, 53]; combinations of diagnoses from at least 2–10 groups of diagnoses. Risk factors are not included in this definition of multimorbidity since they become meaningless to the individual patient if they do not explain possible co-occurring symptoms [54, 55].

### Data collection

The data collection followed a semi-structured interview guide (Interview guide see Additional file 2) with the Needs-based approach as the framework [42]. The interview guide’s first part covered overall themes such as QoL, needs and expectations in life. It encompassed open-ended questions to let the informants talk about experiences with living with multimorbidity concerning their QoL. The second part was flexible and only touched upon if relevant to the informant. It included subthemes as physical ability, self-determination, and mental development. These subthemes were inspired by existing Needs-based questionnaires for patients with single diagnoses, PROMs identified in our systematic review and Maslow’s hierarchy of human needs [39, 42]. The guide was adjusted for subsequent interviews when new themes appeared during an interview. The interviews were conducted mainly by the first author, supervised by all the authors, including the last author, an experienced anthropologist trained in semi-structured interviews. The sampling of informants was guided by information power as the framework, which encourages the use of relatively small samples when high information power of data is obtained [49]. The information power was evaluated continuously throughout the study and determined by considering the specificity of the sample, applied theory, quality of the dialogue and variation of the data in relation to the study’s aim [49]. These topics were discussed among the authors after almost every interview and directed the data collection. The interview phase was ended when we believed each topic was saturated and no new themes emerged. All interviews lasted approximately one hour and were conducted in Danish. After each interview, field notes with observations were written down. The recorded interviews were transcribed, and the selected citations were translated into English.

### Data analysis

The data was thematically analysed by KB and AJ, inspired by Braun and Clarke’s reflexive approach [56, 57], which was suitable with its flexible theoretical and analytical scope. All transcribed interviews were read in full text, taking notes of initial thoughts. Using NVivo we systematically coded the data twice. The first time we coded all aspects occurring across the entire dataset to get familiar with the data. The second coding focussed on semantic and latent meanings and involved identifying what might form repeated patterns (themes) and collating data extracts within each code. The large number of codes that occurred were sorted into code groups based on repeated patterns as a broader level of potential domains, comparing coded data within the themes. This phase was discussed within the interdisciplinary author group. KB repeatedly revisited data and theory through these phases as an abductive process. Finally, after the interview process had ended, recontextualization was conducted by comparing the themes, selected quotes and fieldnotes with the full-text transcriptions to ensure content coverage regarding the material. After that, the final domains were refined and named.

## Results

The informants with multimorbidity talked openly and freely about their QoL, not only in terms of health but as intertwined needs affected by their chronic conditions. We have analysed and categorised the informants’ needs into six domains; “Physical ability”, “Self-determination”, “Security”, “Partner and social life”, “Self-image”, and “Personal finances”. Descriptions of each domain are presented in Table 2.

### Needs among patients with multimorbidity

#### Physical ability

Having a high degree of physical ability was essential for all the informants because it affected all the other needs. Robert, in his early fifties, was extremely limited in his physical ability. Living with chronic pain in his hips because of severe obesity and impaired balance because of sequalae from his diabetes, he always walked with crutches. He had not been out of his first-floor apartment in a subsidised residential construction for three weeks due to a steep doorstep and his recent hospitalisation with an infection.

*"I can't really visit anyone. If I visit you, for instance, I would have difficulties knowing if I would be able to sit down on your chairs, in case they are too low. So I don't just drive out to someone and visit them, because it is so embarrassing to stand there and say, well where do I sit? And so I end up not visiting them. So quite quickly you become very lonely and sort of trapped….''*

Physical ability was stated as a core need for QoL in conversations with the informants. Moreover, as Robert shows by connecting physical ability with his feeling of loneliness, it had implications on other needs. Several informants voiced dilemmas and limitations arising from a lack of physical ability when talking about their QoL.

#### Self-determination

Self-determination was a need raised in close relation to the informants' lack of physical ability. The informants did not necessarily use the word self-determination but spoke of limitations and restrictions caused by their illnesses.

Some informants voiced how a lack of possibilities for self-determination would deprive them of their authority. Instead, they felt their lives would be determined by, for example, illnesses and treatments or local authority employees. Other informants expressed frustrations when trying to take control of their daily life. Daniel was used to an active life full of impulsive activities with his friends, but with his recent diabetes diagnosis, he felt restrained by his treatment with insulin.

"*It is really difficult to make plans (…) Normally I would just go out. If I met some friends saying* "*shall we do this?"–No, I can't.*"

Structure, organising and arranging plans around illnesses and treatments became essential to several informants to feel they were in control and to meet the need for self-determination. Lower QoL relating to lack of self-determination was also apparent among those informants that felt restrained because they had become dependent on family, friends, or healthcare workers.

#### Security

Many informants spoke of worries regarding their illnesses and the need for certainty either for themselves or their close relatives. The worries concerned uncertainties about, for example, treatments or fear of their health conditions worsening. Mary’s psychiatric condition had been well treated for years and gave her no concerns. Nevertheless, having just been diagnosed with diabetes, the combination and possible drug interactions worried her tremendously. Mary felt frustrated and insecure as she experienced there was nowhere she could turn to with these considerations. Other worries concerned the informants' personal finances (see below) or burdening close relatives with illnesses. *"What I'm most afraid of is that I will not be there for the children to sufficiently guide and secure them."*

For James, with three children, imagining not being there for them because of his illnesses was the most horrific worry.

#### Partner and social life

Informants worrying about their close relatives unfolded both the fear of having less ability to support their family and friends and the restrained feeling of being dependent on them. Furthermore, some informants without a partner worried that their need for a partner or romantic relationship could not be fulfilled. They voiced how their illnesses challenged all their social relations towards friends, family, colleagues, and how their illnesses were seen as an obstacle to getting a partner.

Peter felt his loss of physical ability had affected his relationship with his closest family.

*"In the past, I repaired my family’s cars, I helped them with fixing things in their houses and that sort of stuff. But now that I can’t do that anymore, well, then I’m sort of pushed out of the family. (…) that’s actually quite lonely, right?"*

With his back problems followed a severe depression, and he was no longer capable of maintaining his job as a workman. His job had been his identity and this had implications on his social life and self-image.

Robert told how, because of his illnesses, he felt his situation affected his possibilities of getting a partner:

*"You know, sitting down and saying, now I want to have a relationship and a life again. Forget it, Robert, you have nothing to offer (…) I have no financial security, I can't have sex, I can't go out and create experiences, going on holidays together and that sort of thing. I can't really do anything anymore. There is no more to it."*

#### Personal finances

As with physical ability, the informants stated how their personal finances was a fundamental unmet need that impacted other needs. Amanda had retired early because of her illnesses and lived with her grown-up daughter in a rented farmhouse. With her low income, she had not been able to pay her bills the last couple of months and had just received a letter from her power supply company warning her electricity would soon be cut off.

*"Money, you see.. Now I'm about to cry again, but the thing about not having money, that social security benefits cover your rent and then basically nothing else. It does something to your quality of life. (…) money influences, well, very many things in life, right? (…). It's like this pillar everything has to lean on. And it doesn't feel like that right now."*

Amanda makes it very clear that her financial situation was closely related to the feeling of being secure; for others, it was linked to their self-image.

#### Self-image

The informants expressed a need to maintain a self-image which they could be proud of. This was closely linked to their role in society, working identity and formed by how they believed their surroundings saw them.

Robert, a former successful self-employee, was seeking early retirement at the time of the interview because of his physical limitations. Therefore, his need for a self-image he could be proud of was clear:

"*I have always had the feeling that your job is a part of your identity. So everybody who has looked down at me my whole life, because I was fat or whatever, well, they regained their respect when they heard what I earned and what my job was. And then, all of a sudden, I'm not that anymore."*

For James maintaining a job was a part of educating his children by showing them "*to be industrious".* It was of such importance to him that his two children, aged 4 and 12, still believed he went to work every day, even though it was more than two years ago that he was approved for early retirement.

*"I have always been the sort of person that wanted to educate my children, so that my children know morals and ethics (…). I have felt shameful for a long time that I'm ill and can't work. I still do. The background I come from, well, there you work. (…) There are no other possibilities. It is a disgrace not being able to support your family."*

James expressed his need to be a role model, followed by a sense of shame when his illnesses hindered this. Several of the informants gave voice to this shame and a linked feeling of guilt.

## Discussion

This study points to the unmet needs that reduce the QoL of our informants living with multimorbidity analysed into the domains; “Physical ability”, “Self-determination”, “Security”, “Partner and social life”, “Self-image”, and “Personal finances”. Physical ability and personal finances were voiced as core needs that had a high impact on the other needs. It unfolded as a domino effect where for example, a lack of money due to early retirement, because of a limited physical ability, resulted in a low self-image and a belief they would not be able to find a partner. The impact of unmet needs affecting other needs was outlined differently among the informants, yet, all leading back to problems with their physical ability and personal finances due to their illnesses. This domino effect illustrates how HRQoL measures focusing solely on health do not encompass the complexity of living with multimorbidity, even though physical ability was voiced as a core need. The Needs-based approach obliges to this deficiency, as HRQoL is just one aspect of QoL, and physical ability only becomes essential insofar as it does not allow needs to be fulfilled [20].

Where Maslow outlined a hierarchy of needs [39], we argue that patients with multimorbidity experience their QoL affected by *intertwined needs*, the domino effect of unmet needs. The perceived burden on their QoL was not a simple sum of each unmet need, as the problems were intertwined, causing a more complex and heavier impact on their QoL. The Cycle of Complexity Model incorporates existing models on complexity for patients with multiple chronic conditions [58]; among others, it is built upon Shippee's model describing complexity as the imbalance between the patients' workload and capacity [22]. Patients' preferences and expectations are the core of the model whereas the contextual factors (social relations, organisational and community context) has an overall impact on all the complexity domains. Grembowski has further defined complexity for patients with multiple chronic conditions as the misalignment between the individual patient's needs and the healthcare system's capacity to meet these needs [59]. Nevertheless, the specific needs are not defined, but the factors that influence them, such as sex, race and age. This study builds upon these aspects of complexity and emphasises the patients' preferences by bringing out the specific needs relevant to patients living with multimorbidity, defined by themselves.

The pivotal role of the healthcare system's capacity to meet patients' needs stated by Grembowski overlaps with what was voiced by some of the informants from our study. They felt their multimorbidity and its consequences were decisive in terms of how they were met by healthcare professionals, local authorities, as well as friends and family. It was of such importance to the patients that it became a separate topic, which will be discussed in a future article.

As with QoL, the definition of HRQoL is not clear and has evolved to be used synonymously with health, health status, and QoL [18]. It should be noted that with the work of the COSMIN group [60] and the ISO-QoL community [19], much more attention has been given to the importance of patient perspectives and involvement in the development of new QoL and HRQoL measures. However, this does not change the fact, that EQ-5D and SF36 are still the most widely used measures among patients with multimorbidity [6, 7, 9, 25].

The heterogeneity in the measured effects of studies targeted patients with multimorbidity [11–14], we believe, is partly due to the complexity within this croup and the derived DIF. Hence, studies demonstrating low QoL assessments are potentially concluding on invalid results. In our future work, we will test and develop relevant items for MMQ, a PROM specific for patients with multimorbidity. The PROM will be statistically assessed for its validity using modern psychometrics, including DIF assessment.

### Strengths and limitations

The strength of this study is the thorough analyses of needs related to QoL based solely on patients with multimorbidity perspectives. Additionally, the thoroughly considered specifications of the inclusion criteria ensured that we unfolded QoL for the more complex patients, as this is the group, we believe may benefit from healthcare interventions. The interdisciplinary collaboration between medical doctors and an anthropologist provided in-depth knowledge about patients with multimorbidity through several years of clinical experience, long-term ethnographic fieldwork, and research within this group. This can be seen as a strength and a limitation, as our presumptions of the relevance of the Needs-based approach for patients with multimorbidity may have affected the data collection and analysis process. Furthermore, we claim to move beyond a traditional clinical focus on QoL, but it can be discussed if this is feasible, the first author who conducted most interviews being a medical doctor. We have strived to be reflexive about our position and allowed for the patient perspective to guide all study phases.

The inevitable weakness of this study is the heterogeneity of patients with multimorbidity, which raises the question of whether we can generalise on the relatively small sample size of 15 patients in 19 interviews. We argue this is possible due to the purposive sampling ensuring data with high information power [49]. Another limitation is that the needs may vary in other cultural settings. Fundamental needs such as food and water were not touched upon by the informants in this study, which we ascribe to the high standard of living in Denmark. [49]

## Conclusion

This study shows six intertwined overall domains regarding Needs-based QoL relevant for patients with multimorbidity: “Physical ability”, “Self-determination”, “Security”, “Partner and social life”, “Self-image”, and “Personal finances”. Conceptualising Needs-based QoL specifically to this group ensures content validity in the future development of MMQ, a PROM measuring QoL among patients with multimorbidity. “Physical limitations” and “Personal finances” were stated as core needs implicating the other domains. These six domains are relevant in a Danish context, with a generally high standard of living.

## Supplementary Information

**Additional file 1:** Specified inclusion criteria.

**Additional file 2:** Interview guide.

## Data Availability

The dataset, consisting of Danish transcriptions, generated during the current study are not publicly available due to protection of the informants’ anonymity and European General Data Protection Regulation (GDPR) law. Data is available in anonymised form upon reasonable request to the first author. All data were analysed using NVivo12.

## Abbreviations

ADHD: Attention Deficit Hyperactivity Disorder
COPD: Chronic Obstructive Pulmonary Disease
EQ-5D: European Quality of Life–5 Dimensions
HRQoL: Health-Related Quality of Life
MMQ: MultiMorbidity Questionnaire
PROM: Patient-Reported Outcome Measure
PTSD: Post-Traumatic Stress Disorder
QoL: Quality of Life
SF-36: Short Form-36
