# Supplementary material for: Conceptualisation of a measurement framework for Needs-based Quality of Life among patients with multimorbidity
Source: J Patient Rep Outcomes. 2022 Jul 27;6:83. doi: 10.1186/s41687-022-00489-0 (PMC9329502; doi:10.1186/s41687-022-00489-0)
Supplement: Supplementary file 1 — Additional file 1: Specified inclusion criteria. [file 41687_2022_489_MOESM1_ESM.docx]

**Additional File 1**

**Inclusion criteria**

*The following more stringent inclusion criteria were sent to the recruiting general practitioners:*

Informants above the age of 18 years with more than one chronic illness.

More specifically Kristine wishes to interview patients who are suffering, reflective informants who experience problems in relation to their health status because of their multimorbidity.

The aspect of suffering might be expressed by one or more of the following:

The patients have one or more psychiatric diagnosis

And/or is under the age of 65 years

And/or is newly diagnosed with one of their chronic illnesses

And/or is poorly regulated in their treatment regimes

And/or has low compliance

And/or is anxious/neurotic

And/or is low educated

And/or has a weak network

And/or fell neglected by the healthcare system

And/or fell limited physically or socially because of their illnesses

And/or fell unsafe in their everyday lives because of their health status
