# Supplementary material for: Conceptualisation of a measurement framework for Needs-based Quality of Life among patients with multimorbidity
Source: J Patient Rep Outcomes. 2022 Jul 27;6:83. doi: 10.1186/s41687-022-00489-0 (PMC9329502; doi:10.1186/s41687-022-00489-0)
Supplement: Supplementary file 2 — Additional file 2: Interview guide. [file 41687_2022_489_MOESM2_ESM.docx]

**Additional File 2**

**Interview guide***(The original interview guide was translated from Danish)*

We plan to conduct 20-24 semi-structured interviews with informants with multimorbidity; more than one chronic illness. To secure variation, we try to obtain a strategical selection of informants regarding: Age, gender, marital status, educational background, job situation and municipality of residence. Regarding their health status, we aim at a variation of the degree of the chronic illnesses as well as the different combinations of illnesses/risk factors/diagnoses of the individual informant, including psychiatric diagnosis.

*Purpose of the interviews:*To identify items/themes covering quality of life, implicit burden of illness and burden of treatment among patients with multimorbidity.

The interview is constructed so that the informant is invited to talk openly and freely about the theme’s quality of life, needs and expectations, and how their illnesses interact with these concepts in the first open part. This includes questions within the Needs-based categories if they are considered relevant in the conversation.

In the second part of the interview, the informant will be presented with statements within the Needs-based categories and are asked to reflect on whether they find they are related to their quality of life.

*Introduction*Information about:

The interviewer's name and role
 Purpose of the interview and why he/she is selected as an informant
 What themes the interview will include
 How data will be stored, analysed and mediated
 That the informant at any time has the opportunity not to answer questions that he/she does not wish to
That the informant at any time has the opportunity to withdraw from the study

Furthermore, the informant will be informed that there might be questions that the informant hasn’t thought about before and that he/she is welcome to reflect on the theme. No answer is “right” or “wrong”.

The informant fills out a written consent on participation and gets a copy.

***Background***

A brief introduction to the informants’ background: Age, education, marietal status, network, job situation, housing conditions and economy.

***Quality of life as a general concept***

What is quality of life to you?
How do your health/illnesses/treatments interact with your quality of life?

***Expectations***What are your expectations of a good life for you?

How does your health/illnesses/treatments interact with these expectations?

***Needs***

What needs have to be fulfilled for you to have a good life?

How do your health/illnesses/treatments interact with these needs?

The following needs (based on the Needs-based model) can be included according to the informants’ answers to the above themes and if they are relevant to the conversation:

*Subtheme A: Physical ability*

Are there things you can not do because of your illnesses?
(Work/socially/at home/spare time activities)……. Tell me more about that

In what situations?

What is it that limits you?

(How does that influence your quality of life?)

*Subtheme B: Security*

Do you sometimes feel unsafe or worried because of your illnesses or treatments?

(health/personal economy/responsibilities towards others)…. Tell me more about that

What do you do in situations where you feel unsafe?

(How does that influence your quality of life?)

*Subtheme C: Partner/network*

Do you have a partner? If yes – do your illnesses influence your relationship?

How does your network (family and friends) effect your health? Do you get help to handle your illnesses or treatments from your family? Friends? Your general practitioner? Others?

(support from your general practitioner/family/friends concerning illnesses and treatments?

How does your health affect your network (family/friends)? Do your illnesses affect anything in your relationship with your family/friends?

(How does that influence your quality of life?)

(…other patients with more than one chronic illness have talked about…)

*Subtheme D: Self-determination*

Are there any situations where you feel limited because you have to take your illnesses or treatments into account?

(diet/spare time activities/visiting others) …… Tell me more about that

Do you do anything differently than if you had been in good health? (Prioritising differently) (diet/exercise)

Do you have an influence on your health?

(How does that influence your quality of life?)

*Subtheme E: Status (socially/financially)*

Is there anything about your self-image that has changed because of your illnesses?

(Feeling useful at work, for your network, feeling old or weak) …… Tell me more about that

Are there situations where you feel people look at you differently because of your illnesses or treatments? …… Tell me more about that

*Subtheme F: Mental development*

Have your illnesses limited you in growing/knowledge/education/your job situation?

(Hvordan indvirker det på din livskvalitet?)

*Subtheme G:* *Well-functioning society*

Other patients with more than one chronic illness have talked about:

Feeling you are receiving unfair treatment from the health care system

Crucial factors: Considerations towards children, psychiatric diagnosis, war

Approach to life: Getting the best out of it/learning to live with it/one day at a time

What you feel your life is worth

Goals/dreams/future/hope

Adjusting to the situation. Thoughts about the illnesses

Gratefulness

Has anything good come out of the health situation?

***Theme 5: Interview effect***

Do you have anything to add that we haven’t talked about?

What had you imagined we would talk about?

How has it been?

Had it been different if I, as the interviewer, hadn’t been a doctor?

Why did you say yes to participating?

Is there anything I should do differently in my following interviews?

*At parting*

Offer contact information to the interviewer

*Finishing revisions*

Immediate impressions are written down:

Central themes for the informant

The interviewer’s impression of the informant

Evaluation of the role of interviewer and the relation to the informant

Overall impression and evaluation of the interview

What went well/not so well

What was included/forgotten?
